# A Novel Wearable Sensor-Based Human Activity Recognition Approach Using Artificial Hydrocarbon Networks

**DOI:** 10.3390/s16071033

**Published:** 2016-07-05

**Authors:** Hiram Ponce, María de Lourdes Martínez-Villaseñor, Luis Miralles-Pechuán

**Affiliations:** Faculty of Engineering, Universidad Panamericana, Mexico City 03920, Mexico; lmartine@up.edu.mx (M.d.L.M.-V.); lmiralles@up.edu.mx (L.M.-P.)

**Keywords:** artificial organic networks, artificial hydrocarbon networks, robust human activity recognition, supervised machine learning, wearable sensors, noise tolerance

## Abstract

Human activity recognition has gained more interest in several research communities given that understanding user activities and behavior helps to deliver proactive and personalized services. There are many examples of health systems improved by human activity recognition. Nevertheless, the human activity recognition classification process is not an easy task. Different types of noise in wearable sensors data frequently hamper the human activity recognition classification process. In order to develop a successful activity recognition system, it is necessary to use stable and robust machine learning techniques capable of dealing with noisy data. In this paper, we presented the artificial hydrocarbon networks (AHN) technique to the human activity recognition community. Our artificial hydrocarbon networks novel approach is suitable for physical activity recognition, noise tolerance of corrupted data sensors and robust in terms of different issues on data sensors. We proved that the AHN classifier is very competitive for physical activity recognition and is very robust in comparison with other well-known machine learning methods.

## 1. Introduction

The interest in human activity recognition research has been growing in context-aware systems for different domain applications. Human activity recognition (HAR) deals with the integration of sensing and reasoning in order to better understand people’s actions. Research related to human activity recognition has become relevant in pervasive and mobile computing, surveillance-based security, context-aware computing, health and ambient assistive living. Recognizing body postures and movements is especially important to support and improve health systems, as discussed below.

In their survey, Avci et al. [1] reviewed several medical applications of activity recognition for healthcare, wellbeing and sports systems. Regarding medical applications using HAR with wearable sensors, the authors report examples in the literature of healthcare monitoring and diagnosis systems; rehabilitation; systems to find correlation between movement and emotions; child and elderly care. They also reviewed assisted living and home monitoring systems improving the quality life and ensure the health, safety and wellbeing of children, the elderly and people with cognitive disorders. The authors also state that numerous activity recognition systems using wearable sensors have been proposed for sports and leisure applications; for example: daily and sport activity recognitions; detection of motion sequences in martial arts to increase interaction in video games or martial arts education; monitoring sport activities in order to train and monitor the performance.

Preece et al. [2] reported activity classification systems to find links between common diseases and levels of physical activity. The authors also reviewed systems that provide information on daily activity patterns to improve the treatment and diagnosis of neurological, degenerative and respiratory disorders. Other reported systems quantify levels of physical activity providing feedback and motivating individuals to achieve physical activity goals. Guidoux et al. [3] presented an approach based on smartphone sensors for estimating energy expenditure recognizing physical activities in free-living conditions. In summary, health systems and assistive technologies can benefit from activity recognition and deliver personalized services.

Automated human activity recognition is a challenging task. Two main approaches are used to perform the task of activity recognition: vision-based and sensor-based activity recognition [4,5]. The vision-based approach is based on image processing of video sequences or digital visual data provided by cameras. No wearable or smartphone sensors are required, but it depends on the image quality. The quality of cameras, lighting and environments, among others, are factors that determine image quality. Visually monitoring the actor behavior entails privacy issues. The sensor-based approach is focused on activity monitoring using wearable [5], smartphone sensors and technologies [6] or object embedded sensors [7]. There are several drawbacks in these approaches: wearing sensors or smartphones for a long period of time is necessary, and there might be battery issues. However, the main problem when using sensor-based approaches is the different types of noise found in input features due to sensor errors or noisy environments. The output class can also have errors. Noise in data hampers the human activity recognition classification process.

Nettleton et al. [8] state that “machine learning techniques often have to deal with noisy data, which may affect the accuracy of the resulting data models.” This statement is also true in the activity recognition classification process given the great variations on the types, number and positioning of sensors. Sensor characteristics change also across different subjects and for the same individual [2]. Therefore, in order to develop a successful activity recognition system, it is necessary to use stable and robust machine learning techniques capable of dealing with noisy data.

In this paper, we present a novel machine learning technique to the human activity recognition community: artificial hydrocarbon networks (AHN). Our artificial hydrocarbon networks approach is suitable for physical activity recognition, the noise tolerance of corrupted data sensors and is robust in terms of different issues for data sensors. With the purpose of proving the aforementioned characteristics of our technique, a comparison analysis was performed with the most commonly-used supervised classification techniques in the HAR community. The performance of the proposed AHN classifier was compared to fourteen supervised techniques frequently used in the activity recognition classification process and reviewed in the literature [2,9,10,11].

In order to evaluate the performance of the artificial hydrocarbon network-based classifier, four experiments were designed using the public Physical Activity Monitoring dataset (PAMAP2) [12,13]. The first experiment was done using the entire raw dataset. The second experiment was made performing a prior feature reduction using the recursive feature elimination (RFE) method. The third experiment evaluated noise tolerance in all supervised classifiers using three levels of noise: 7%, 15% and 30%. Noise was simulated with random insertion in input features of the testing set. Lastly, a majority voting across windows-based approach for an HAR system using the proposed method was implemented.

Our results show that the AHN classifier is very competitive for physical activity recognition and is very robust in comparison with the other methods. In addition, this paper also contributes with a benchmark between fifteen supervised machine learning methods in the human activity recognition field, comparing them in terms of: accuracy, macro- and micro-averaging sensibility, precision and $F_{1}$-score and training time; also contrasting the experimental results with recent literature. Notice that the proposed method is not working in real time, and the introduction of the artificial hydrocarbon networks in real-time HAR systems is out of the scope of this work.

The rest of the paper is as follows. Section 2 describes the state-of-the-art sensor-based human activity recognition and discusses noise in the classification process. Then, Section 3 introduces the artificial hydrocarbon networks technique as a supervised learning method, and Section 4 describes our proposal for using an AHN-based classifier in human activity recognition. In order to prove the proposed classifier, a case of study in physical activity monitoring is presented and described in Section 5. In addition, Section 6 presents the results and a discussion of the proposal, as well as a comparison between fourteen supervised classifiers used in HAR. Lastly, Section 7 concludes the paper and highlights future work in this context.

## 2. Sensor-Based Human Activity Recognition

Recognizing a human activity in a wearable sensor-based approach means that: (i) the activity is present in the physical environment; (ii) sensors are able to provide a reliable representation of the physical parameters of the environment affected by the activity; and (iii) a classification algorithm recognizes accurately an activity [14]. In that sense, this work is focused on the latter component of the wearable sensor-based human activity recognition approach.

Currently, many learning methods have been used in recent years for human activity recognition. Several reviews have been published analyzing the performance of different classifiers in the human activity recognition research area for applications in home care systems, surveillance, physical therapy and rehabilitation, sports improvement, among others. The literature reports several surveys and comparisons for sensor-based human activity, like in [1,2,4,9,11,15,16,17], and vision-based human activity recognition can be found in [18,19].

Since our work is mainly focusing on sensor-based human activity recognition and data-driven approaches, this section is particularly interested in reviewing related works regarding the stability and robustness of machine learning techniques when confronted with the task of human activity recognition. Thus, noise in the human activity recognition classification process is discussed, firstly. Subsequently, related works on machine learning techniques used for human activity recognition are reviewed.

### 2.1. Noise in the Human Activity Recognition Classification Process

A classification process must be done in order to recognize human activity given that the activity is present and wearable sensors reliably represent physical parameters affected by the activity. The goal of the classification task for human activity recognition is to interpret the features of physical parameters and perform a correct classification of the activity [14].

Noisy data are often provided in machine learning processes, making it more difficult to obtain accurate models for real problems [8]. Different types of noise can also be found in the human activity recognition classification process. Input features may have noise for several reasons, such as: (1) sensor miscalibration; (2) dead or blocked sensors; (3) errors in sensor placement; (4) activities registered in noisy environments; (5) activities interleaved, so that the events are not only related to one activity. Classification labels in the output class need human intervention, and it is therefore likely to have errors, as well. As in other classification problems, noise can be located in training and/or test data.

It is difficult to measure the impact of each type of noisy data in the classification process. Nettleton et al. [8] reviewed works that studied the impact of noise for several learners and presented a comparison of the effect of attribute and class noise on models created by naive Bayes, C4.5 decision tree, an instance-based algorithm, and support vector machines. They compared the techniques’ performance with thirteen classification problems (activity recognition is not included). In the latter work, the authors proved that naive Bayes is relatively more robust to noisy data than the other three techniques, and SVM presented the poorest performance. In this regard, we agree with Nettleton et al. [8] on two statements:

1. Developing learning techniques that effectively and efficiently deal with noisy types of data is a key aspect in machine learning.
2. There is a need for comparisons of the effect of noise on different learning paradigms.

These two statements are also pertinent for human activity recognition domain.

### 2.2. Machine Learning Techniques Used for Human Activity Recognition

The growing interest on human recognition and the great advances in sensor technologies create the necessity for developing robust machine learning systems. Applications in the field of activity recognition need to deal with a large number of mult imodal sensors that provide high-dimensional data with large variability; thus, data may be missing, and labels can be unreliable.

Recently, some efforts have been done to promote the development of robust machine learning techniques, especially in the domain of activity recognition. The workshop on robust machine learning techniques for human activity recognition is one example of these efforts [20]. An overview of activity recognition describing the major approaches, methods and tools associated with vision and sensor-based recognition was presented by Chen et al. [4]. In fact, the authors made the distinction between data-driven and knowledge-driven approaches. The sensor-based approach is focused on activity monitoring using wearable or smartphone sensors and technologies, while the vision-based approach requires image processing of video sequences or digital visual data provided by cameras [21].

Preece et al. [2] present an introduction and research review of different machine learning techniques used for human activity recognition and their failures. Currently,the authors discuss findings and results obtained with the following learning techniques used in activity classification: threshold-based classification, hierarchical methods, decision trees, *k*-nearest neighbors, artificial neural networks, support vector machines, naive Bayes and Gaussian mixture models, fuzzy logic, Markov models, combined classifiers and some unsupervised learning methods. They made a summary of studies comparing different classifiers and an overview of the advantages and drawbacks of each of the aforementioned methods. Their comparison includes the number and type of activities classified, accelerometer placements and inter-subject classification accuracy. From this overview, we extract and highlight the following statements [2]:

- “The variability in activities, sensors and features means that it is not possible to directly compare classification accuracies between different studies.”
- “... there is no classifier which performs optimally for a given activity classification problem.”
- “... there is a need for further studies investigating the relative performance of the range of different classifiers for different activities and sensor features and with large numbers of subjects.”

Regarding noise, Preece et al. [2] only mentioned wavelet analysis techniques for suppressing noise, but they had not mentioned anything about the classifier’s robustness or stability.

Dohnálek et al. [11] present a comparison of the performance only in terms of the accuracy of several classifiers: two orthogonal matching pursuit techniques, *k*-nearest neighbors, classification and regression tree (CART) techniques and global merged self-organizing maps. Their dataset contains data of sensors that measure temperature and 3D data from the accelerometer, gyroscope and magnetometer of nine healthy human subjects. Their results confirm that a compromise between speed and accuracy must be made given that the best classifiers are slower than the worst. It is important to notice that only a brief discussion of time complexity was presented, and no discussion regarding the robustness of the classifiers was done.

Lara et al. presented a summary of classification algorithms used in human activity recognition systems in their survey [9]. They discussed the advantages and limitations of different types of classifiers: decision trees, Bayesian instance-based artificial neural networks, domain transform, fuzzy logic, regression methods, Markov models and classifier ensembles. In addition to this work, the authors did not mention the impact of noise in the process of activity recognition; however, Lara presented experiments addressing this impact in his dissertation [22]. He induced noise by arbitrarily modifying the labels in the dataset to assess the effectiveness of the proposed probabilistic strategies. His results show that some classification algorithms are more tolerant to noise than others.

Lustrek et al. [23] compared the performance of eight machine learning techniques in fall detection and activity recognition. They added Gaussian noise to their input recordings of body tags to the shoulders, elbows, wrists, hips, knees and ankles. They presented classification accuracy results for clean and noisy data in support vector machines, random forest, bagging and AdaBoost classifiers. The best accuracy (support vector machines) on clean data was 97.7% and on noisy data 96.5%.

Ross et al. [24] presented a comparative analysis of the robustness of naive Bayes, support vector machines and random forest methods for activity with respect to sensor noise. The authors performed experiments with collections of test data with random insertions, random deletions and dead sensors. They simulated miscalibrated and dead sensors. Random forest models outperform the other methods in all of their experiments. In their brief study, the three chosen methods were consistent in their relative performance.

To this end, the Opportunity Activity Recognition Challenge was set to provide a common platform to allow the comparison of different machine learning algorithms on the same conditions. Chavarriaga et al. [25] presented the outcome of this challenge. They reported the performance of the following standard techniques over several subjects and recording conditions: *k*–nearest neighbors, nearest centroid classifier, linear discriminant analysis and quadratic discriminant analysis. One of the subjects had different sensor configurations and noisy data. The dataset used for the challenge is a subset of the one presented by Roggen et al. in [10]. These efforts provide a method of comparison of machine learning techniques using common benchmarks.

## 3. Artificial Hydrocarbon Networks as a Supervised Learning Method

Nature-inspired computing promotes methodologies, techniques and algorithms focusing on the computation that takes place in nature [26]. Moreover, in machine learning, heuristic- and meta-heuristic-based methods have been widely explored in order to efficiently tackle real-life problems that are difficult to solve due to their high complexity and limitations of resources to analyze and extract experience from them [26]. Recent works have introduced artificial hydrocarbon networks as a supervised learning algorithm [27], which we use as a classifier for human activity recognition. Thus, this section briefly describes the high-level framework of artificial hydrocarbon networks, called artificial organic networks, and then a full description of the artificial hydrocarbon networks algorithm and its characteristics is exposed.

### 3.1. Artificial Organic Networks

The artificial organic networks (AON) technique is a machine learning framework that is inspired by chemical organic compounds [27], such that all definitions and heuristics are based on chemical carbon networks. Currently, this technique proposes two representations of artificial organic compounds: a graph structure representing physical properties and a mathematical model behavior representing chemical properties.

The main characteristic of the AON framework is that it packages information into modules, so-called molecules [27]. Similar to chemical organic compounds, artificial organic networks define heuristic mechanisms for generating organized and optimized structures based on chemical energy. In a nutshell, artificial organic networks allow [27]: modularity, inheritance, organizational and structural stability.

Currently, artificial organic networks define a framework in order to develop useful learning algorithms inherit to it [27], as shown in Table 1. Reading bottom-up, the first component of this framework defines the basic units that can be used in the machine learning algorithm, the second level is related to the interactions among components to compute nonlinear relationships. Then, the third level of the framework refers to the chemical heuristic rules that control the interactions over components. These three levels are also mathematically modeled in terms of their structure and functionality, and lastly, the implementation level considers training learning models and then inferring from them [27,28]. Detailed information of the AON-framework can be found in [27,28].

### 3.2. Artificial Hydrocarbon Networks Algorithm

Artificial hydrocarbon networks (AHN) algorithm is a supervised learning algorithm with a graphical model structure inspired by chemical hydrocarbon compounds [27]. Similar to chemical hydrocarbon compounds, artificial hydrocarbon networks are composed of hydrogen and carbon atoms that can be linked with at most one and four other atoms, respectively. Actually, these atomic units interact among themselves to produce molecules. Particular to this method, the basic unit with information is the CH-molecule. It is made of two or more atoms linked between each other in order to define a mathematical function φ centered in the carbon atom and parameterized with hydrogen-based values attached to it, as shown in Equation (1); where φ∈R represents the behavior of the CH-molecule, *σ* is a real value called the carbon value, $H_{i}\in C$ is the *i*-th hydrogen atom linked to the carbon atom, *k* represents the number of hydrogen atoms in the molecule and *x* is the input to that molecule [27,29,30].

(1) $$\varphi \left(x\right)=\sigma \prod_{i=1}^{k\leq 4}\left(x-H_{i}\right)$$

If a CH-molecule is unsaturated (i.e., k<4), then it can be joined together with other CH-molecules, forming chains of molecules, so-called artificial hydrocarbon compounds. In [29,30,31], the authors suggest using saturated and linear chains of molecules like in Equation (2); where $CH_{k}$ represents a CH-molecule with *k* hydrogen atoms associated with it, and the line symbol represents a simple bond between two molecules. Notice that outer molecules are $CH_{3}$, while inner molecules are $CH_{2}$.

(2) $$CH_{3}-CH_{2}-\cdots -CH_{2}-CH_{3}$$

Artificial hydrocarbon compounds also have associated a function *ψ* representing their behavior. For instance, the piecewise compound behavior [27] ψ∈R can be expressed as Equation (3); where $L_{t}$ represents the *t*-th bound that limits the action of a CH-molecule over the input space. In that sense, if the input domain is in the interval $x\in [L_{min},L_{max}]$, then $L_{0}=L_{min}$ and $L_{n}=L_{max}$, and the *j*-th CH-molecule acts over the interval $\left[L_{j-1},L_{j}\right]$, for all j=1,...,n.

(3) $$\psi \left(x\right)=\left\{\begin{array}{cc} \varphi_{1}\left(x\right) & L_{0}\leq x<L_{1}\\ \cdots & \cdots \\ \varphi_{n}\left(x\right) & L_{n-1}\leq x\leq L_{n} \end{array}\right.$$

To obtain the bounds $L_{t}$ for all t=0,...,n, a distance *r* between two adjacent bounds, i.e., $\left[L_{t-1},L_{t}\right]$, is computed as in Equation (4); where *r* represents the intermolecular distance between two adjacent molecules. In addition, Δr is computed using a gradient descent method based on the energy of the adjacent molecules ($E_{j-1}$ and $E_{j}$) like in Equation (5), where 0<η<1 is a learning rate parameter [27,28,31]. For implementability, the energy of molecules can be computed using a loss function [27].

(4) r=r+Δr

(5) $$\Delta r=-\eta (E_{t-1}-E_{t})$$

At last, artificial hydrocarbon compounds can interact among themselves in definite ratios forming a mixture S∈R. For this method, weights are called stoichiometric coefficients, and they are represented as elements $\alpha_{i}\in R$, as shown in Equation (6); where *c* is the number of compounds in the mixture [27]. For this work, the artificial hydrocarbon networks structure considers one compound, such that, c=1 and $S\left(x\right)=\psi_{1}\left(x\right)$.

(6) $$S\left(x\right)=\sum_{i=1}^{c}\alpha_{i}\psi_{i}\left(x\right)$$

**Figure 1:**
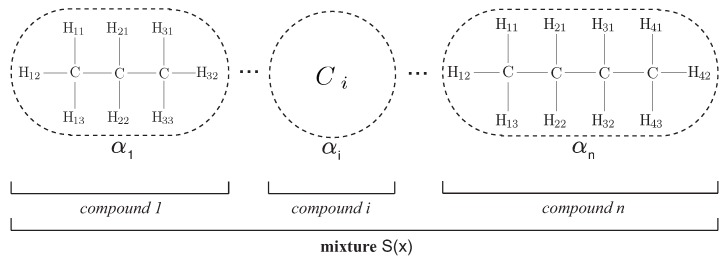
Structure of an artificial hydrocarbon network using saturated and linear chains of molecules [29]. For this work, the topology of the proposed classifier considers just one hydrocarbon compound (see Section 4).

Formally, an artificial hydrocarbon network is a mixture of artificial hydrocarbon compounds (see Figure 1), each one obtained using a chemical-based metaheuristic rule. The training algorithm is known as the AHN-algorithm [28,29,30]; and for this work, the AHN-algorithm was reduced to Algorithm 1 for saturated and linear hydrocarbon compounds. Notice that Algorithm 1 reflects the restrictions about a saturated linear chain of molecules and a piecewise compound behavior imposed for this work. For a detailed description of the general AHN-algorithm, see [27]; and for the implementability, see [28]. In addition, a numerical example of training and testing AHN is summarized in Appendix A. **Algorithm 1** AHN algorithm for saturated and linear hydrocarbon compounds.**Input:** a training dataset Σ=(x,y), the number of molecules in the compound n≥2 and a tolerance value ϵ>0.**Output:** the saturated and linear hydrocarbon compound AHN.Initialize an empty compound AHN={}.Create a new saturated linear compound *C* like Equation (2).Randomly initialize intermolecular distances $r_{t}$.**while**
|y-ψ|>ϵ
**do** Determine all bounds $L_{t}$ of *C* using $r_{t}$. **for each**
*j*-th molecule in *C*
**do**    Determine all parameters of behavior $\varphi_{j}$ in Equation (1) using an optimization process. **end-for** Build the compound behavior *ψ* of *C* using Equation (3). Update intermolecular distances using Equations (4) and (5).**end-while**Update AHN with *C* and *ψ*.**return**
AHN

### 3.3. Characteristics of Artificial Hydrocarbon Networks

The artificial hydrocarbon networks algorithm has some characteristics that can be useful in regression and classification problems. In particular, for this work, both monitoring and noise tolerance tasks in human activity recognition are considered. Thus, several characteristics of AHN related to these tasks are discussed below:

- *Stability*: This characteristic considers that the artificial hydrocarbon networks algorithm minimizes the changes in its output response when inputs are slightly changed [27]. This is the main characteristic that promotes using AHN as a supervised learning method.
- *Robustness*: This characteristic implies that artificial hydrocarbon networks can deal with uncertain or noisy data. The literature reports that AHN can deal with noisy data as it filters information, e.g., AHN has been used in audio filtering [27,31]. Additionally, ensembles of artificial hydrocarbon networks and fuzzy inference systems can also deal with uncertain data, for example in intelligent control systems, like in [29,30].
- *Metadata*: Molecular parameters like bounds, intermolecular distances and hydrogen values can be used as metadata to partially understand underlying information or to extract features. In [27], it reports that the artificial organic networks method packages information in several molecules that might be interpreted as subsystems in the overall domain. For example, these metadata have been used in facial recognition approaches [27].

## 4. Artificial Hydrocarbon Networks-Based Classifier for HAR Systems

From above, this work considers training and using an AHN classifier exploiting stability and robustness characteristics in the field of human activity recognition based on wearable sensors, with particular approaches in monitoring and noise tolerance. Previous work in this direction can be found in [21].

**Figure 2:**
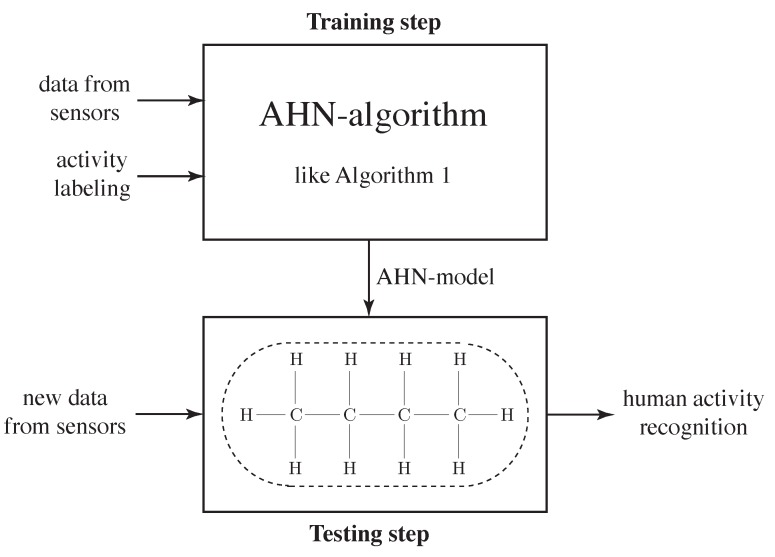
Diagram of the proposed artificial hydrocarbon network-based classifier (AHN classifier). First, data from sensors and activity labeling are used for training the AHN-model, then it is used as the AHN classifier in the testing step.

In this paper, we propose to build and train an artificial hydrocarbon network for a supervised learning classifier (AHN classifier) aiming to monitor human activities based on wearable sensor technologies. In fact, this AHN classifier is computed and employed in two steps: training-and-testing and implementation, as shown in Figure 2.

Currently, the AHN classifier considers that sensor data have already processed in *N* features $x_{i}$ for all i=1,...,N and have organized in *Q* samples, each one associated with its proper label $y_{j}$ representing the *j*-th activity in the set of all possible activities *Y* for j=1,...,J; where *J* is the number of different activities in the dataset. Thus, samples are composed of features and labels as (N+1)-tuples of the form ${\left(x_{1},...,x_{N},y_{j}\right)}^{q}$ for all q=1,...,Q.

Considering that there is a dataset of *Q* samples of the form defined above, then the AHN classifier is built and trained using the AHN algorithm shown in Algorithm 1. It is remarkable to say that this proposal is using a simplified version of artificial hydrocarbon networks. Thus, the AHN classifier is composed of one saturated and linear hydrocarbon compound, i.e., no mixtures were considered (see Figure 1 for a hydrocarbon compound reference). In that sense, the inputs of the AHN-algorithm are the following: the training dataset *Σ* is a subset of *R* samples, from the original dataset, as Equation (7); the number of molecules *n* in the hydrocarbon compound is proposed to be the number of different activities in the dataset (n=J); and the tolerance value *ϵ* is a small positive number selected manually. Notice that the number of molecules in the compound is an empirical value; thus, no pairing between classes and molecules occurs. At last, the AHN-algorithm will compute all parameters in the AHN classifier: hydrogen and carbon values, as well as the bounds of molecules.

(7) $$\Sigma =\left[\begin{array}{c} {\left(x_{1},\ldots ,x_{N},y_{j}\right)}^{1}\\ \vdots \\ {\left(x_{1},\ldots ,x_{N},y_{j}\right)}^{R} \end{array}\right]$$

For testing and validating the AHN classifier, the remaining samples *P* from the original dataset (i.e., such that Q=P+R) form the testing dataset. Then, the testing dataset is introduced to the AHN classifier, previously computed. Lastly, the validation of the classifier is calculated using some metrics (see Section 5). Moreover, new sample data can be also used in the AHN classifier for recognizing and monitoring a human activity based on the corresponding features.

## 5. Case Study: Physical Activity Monitoring Using Artificial Hydrocarbon Networks

In this section, a case study on physical activity monitoring is presented and described in order to prove the performance of the proposed AHN classifier in terms of both monitoring and noise tolerance tasks. In particular, this case study uses a public dataset, and it compares the performance of the AHN-algorithm among other well-known supervised classifiers in the field of human activity recognition. At last, several metrics for classification tasks are also described.

### 5.1. Dataset Description

**Figure 3:**
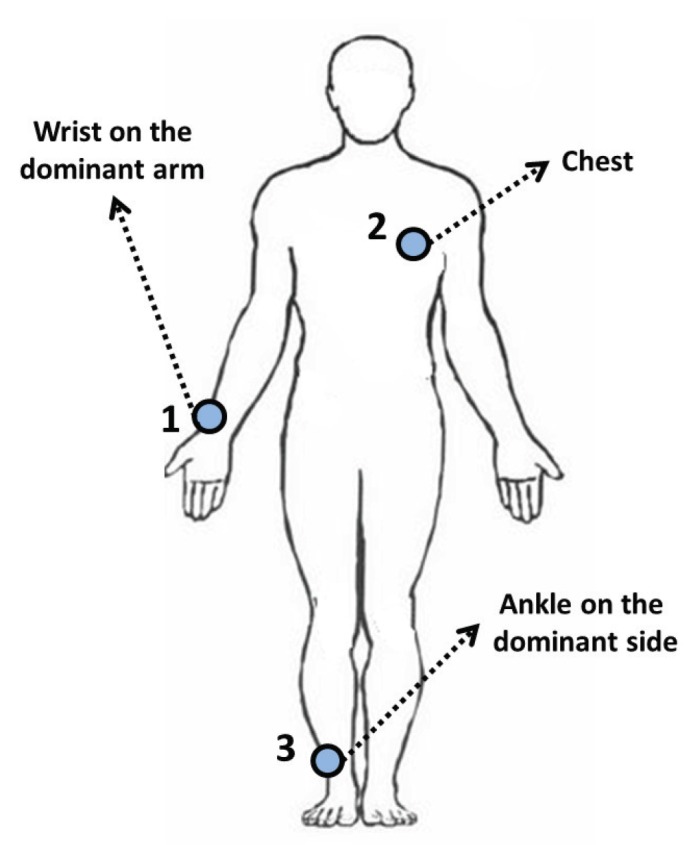
Location of the three wearable sensors used in the dataset (hand, chest and ankle), adapted from [21].

This case study employs the public Physical Activity Monitoring Data Set (PAMAP2) [12,13], which consists on 3,850,505 samples of raw signals from inertial sensors. Those samples were collected from three sensors placed on nine 27-year average people (eight men and one woman), as shown in Figure 3. The subjects performed 18 different activities during intervals of 10 h. However, only eight hours are dedicated to the activities, and the remaining two hours are dedicated to rest and change from one activity to another. Notice that resting and transitional period activities were labeled with zero-value in this dataset. In particular to our case study, we eliminated these zero-labeled activities. Then, the 18 different activities in our modified dataset are summarized in Table 2.

Since the PAMAP2 dataset consists of several measurements from inertial sensors and a heart rate monitor, this case study only considers numerical features from inertial sensors. Each “Colibri” wireless sensor has a total of 17 features: one for temperature, three 3D-acceleration data in inertial measurement units (IMU) sampled at 100 Hz at the scale of Å ± 16 g (13-bits), three 3D-acceleration data (IMU) sampled at 100 Hz at the scale of Å ± 6 g (13-bits), three 3D-gyroscope data (rad/s), three 3D-magnetometer data (*μ*T) and three orientation values. Furthermore, the timestamp was eliminated from the dataset, since it might cause overfitting in supervised classifiers.

To this end, the dataset for the case study is composed of the following samples: 10,200 training samples (600 random samples for each of the first twelve activities and 500 random samples for each of the other activities) and 5100 testing samples (300 random samples for each of the first twelve activities and 250 random samples for each of the remaining activities) chosen randomly from the original dataset. In both cases, samples with missing values were avoided. Notice that since random selection was done, samples in the training and testing sets are not time dependent.

### 5.2. Methodology for Building Supervised Models

In order to prove that our AHN classifier is very competitive for physical activity recognition in terms of performance and noise tolerance, we choose to compare fourteen supervised classifiers, and we conduct three experimental cases.

The supervised classifiers used are the following: stochastic gradient boosting (SGB), AdaBoost (AB), C4.5 decision trees (DT), rule-based classifier (RBC), support vector machines with linear kernel (SVM-L), support vector machines with basis function kernel (SVM-BF), random forest (RF), *k*-nearest neighbors (KNN), linear discriminant analysis (LDA), mixture discriminant analysis (MDA), multivariate adaptive regression splines (MARS), naive Bayes (NB), multilayer feedforward artificial neural networks (ANN) and nearest shrunken centroids (NSC). Selection criteria of these techniques are supported in the reviewed literature [2,9,10,11].

In addition, the methodology also considers a cross-validation technique (10 folds and five repetitions) for each classifier in order to build suitable supervised models. For this case study, the accuracy metric was employed within the cross-validation technique to select the best model for each classifier. Table 3 summarizes the configuration parameters for training these models, using the caret package in R. Notice that the *configurations* column represents the number of different configurations created automatically in the cross-validation technique before selecting a suitable classifier.

On the other hand, each stage of the activity recognition chain (ARC) described by Bulling et al. [32] (i.e., stages from data acquisition, signal preprocessing and segmentation, feature extraction and selection, training and classification) directly influences the overall recognition performance of an HAR system [32]. In particular, feature extraction and selection are common practice to improve the performance of most HAR systems. Hence, if bad design decisions are made, the processed dataset might contain redundant or irrelevant information [9]; the computational demand may unnecessarily increase and also reduce the accuracy of some classification methods [2]. Therefore, some authors choose to experiment with raw data for comparison and evaluation of the recognition performance of supervised and/or unsupervised machine learning techniques [32,33].

**Figure 4:**
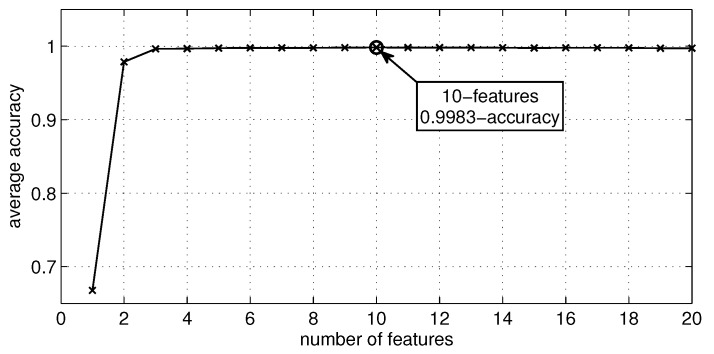
Average accuracy of models with respect to the number of features selected using RFE.

In our work, we choose to compare the following cases trying to minimize the influence of feature generation and extraction:

- *Case 1:* This experiment occupies the raw dataset of the case study as the feature set in order to measure the classification, recognition and monitoring performance over physical activities of all supervised methods, as explained above [32,33].
- *Case 2:* This experiment conducts a feature reduction over the feature set of the previous case, using the well-known recursive feature elimination (RFE) method [34,35]. Table 4 shows the ten retained features, and Figure 4 shows the accuracy curve of its validation. In fact, this experiment aims to compute human activity recognition with the minimal set of raw signals from the sensors’ channels, since minimizing the number of sensors and the usage of their channels is a challenging problem in HAR [9]. The features retained from the initial set of features by the automatic RFE method can apparently contain presumably redundant features (e.g., accelerometers 16 g and 6 g) or some variables that presumably can lead to overfitting. Regarding these two concerns, Guyon et al. [35] proved with simple examples that “noise reduction and consequently better separation may be obtained by adding variables that are presumably redundant” [35]. Thus, sometimes, variables that are apparently redundant, as in our case, can enhance the prediction power when they are combined. At last, the same measures of classification, recognition and monitoring performance were computed.
- *Case 3:* This experiment evaluates noise tolerance in all supervised classifiers using noisy datasets. For instance, Zhu and Wu [36] describe different types of noise generation: in the input attributes and/or in the output class; in training data and/or in test data; in the most noise-sensitive attribute or in all attributes at once. Thus, we decided to generate noise only in some input feature values of some samples of the testing dataset. In order to add noise in a numeric attribute, the authors in [36] suggest selecting a random value that is between the maximal and the minimal values of the feature. For our experimentation, we first randomly removed some feature values using a 7%, 15% and 30% data selection in order to simulate missing values and then automatically replaced the *null* values with the mean of the related feature, as some data mining tools suggest [37]. In fact, this method can be considered as random noise insertion, given that generated missing data are replaced with a value. Notice that supervised models built for this experiment are the same classifiers as those built in the first experiment.

**Figure 5:**
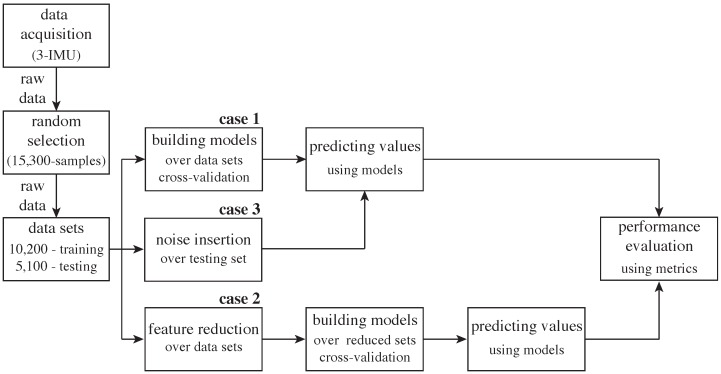
Methodology of experimentation.

The overall methodology is shown in Figure 5. The experiments were executed in a computer Intel^®^Core™i5-2400 with CPU at 3.10 GHz and 16 GB-RAM over Windows 7 Pro, Service Pack 1 64-bit operating system.

To this end, we conduct another experiment using a majority voting across windows-based approach and the AHN classifier to simulate the data flow in a real HAR system and to validate the performance of the proposed classifier in that situation. In fact, we select the first 30 s of each activity carried out by all of the subjects as the testing set, using the same models obtained in the first experiment. Table 5 shows the activities performed by each subject for at least 30 s [12,13]. Then, we apply a fixed window of 2.5 s in size (i.e., 250 samples) without overlapping during the 30 s of each activity. Lastly, a majority voting strategy [32] was employed inside the window in order to finally output the recognized activity. For this experiment, we build the models with the same strategy as followed in the previous cases.

### 5.3. Metrics

This case study uses different metrics to evaluate the performance of the AHN classifier in comparison with the other supervised classifiers, such as: accuracy, sensitivity, precision and $F_{1}\;score$ [38]. In addition, the metrics distinguish two ways of computation: macro-averaging (*M*) and micro-averaging (*μ*) [38]. The first one treats all classes equally, while the second one considers the size of each class. Thus, macro-averaging is important to measure the overall classification, and micro-averaging computes the performance of classifiers in a precise way. To this end, $F_{1}\;score$ was calculated using Equations (8) and (9) [38], respectively.

(8) $$F_{1}\;score_{M}=2\times \frac{precision_{M}\times sensitivity_{M}}{precision_{M}+sensitivity_{M}}$$

(9) $$F_{1}\;score_{\mu }=2\times \frac{precision_{\mu }\times sensitivity_{\mu }}{precision_{\mu }+sensitivity_{\mu }}$$

Additionally, other metrics on the classifiers are computed as well: training_time specifies the training time (in seconds) to build and train a model, and testing_time specifies the evaluation time of an input sample (in milliseconds).

## 6. Experimental Results and Discussion

As said above, three experiments were conducted in order to evaluate the performance in both monitoring and noise tolerance tasks using an artificial hydrocarbon networks-based classifier in the context of the case study previously presented. In addition, a fourth experiment was conducted using a majority voting across windows-based strategy to simulate the data flow in a real HAR system and to validate the performance of the AHN classifier in that situation. Thus, this section presents and analyzes the comparative results obtained in this regard.

### 6.1. Comparative Analysis on Physical Activity Monitoring

To evaluate the performance on monitoring physical activities using the AHN classifier, two experiments were conducted. The first experiment considers the complete dataset of the case study, and the second experiment occupies the reduced dataset using the RFE technique (see Section 5). Table 6 and Table 7 show comparative results (sorted in descending order by accuracy) of the supervised classifiers in terms of the metrics already defined above.

In both cases, the AHN classifier ranks over the mean accuracy, and it is positioned in the first quartile of the evaluated classifiers. Using the complete dataset, the AHN classifier is placed close to decision tree (first place), rule-based (second place) and support vector machine (fourth and fifth places) -based classifiers, as seen in Table 6. In addition, Table 7 shows that the AHN classifier is placed close to stochastic gradient boosting (first place), AdaBoost (third place), random forest (fourth place) and rule-based (fifth place) classifiers.

For instance, the decision tree-based classifier (the best ranked method in Table 6) is 0.52% over the AHN classifier based on the accuracy, and in terms of $F_{1}$-${\mathrm{score}}_{\mu }$, the decision tree-based classifier is 0.33% over the AHN classifier. Using the same comparison, Table 7 shows that stochastic gradient boosting (the best ranked method) is 1.5% and 0.86% over the AHN classifier based on accuracy and $F_{1}$-${\mathrm{score}}_{\mu }$, respectively.

Comparing Table 6 and Table 7, the performance of the methods is modified. For example, the decision tree-based classifier goes down 3.12% in accuracy and 1.35% in $F_{1}$-${\mathrm{score}}_{\mu }$; while stochastic gradient boosting goes up 1.77% in accuracy and 0.57% in $F_{1}$-${\mathrm{score}}_{\mu }$. In this regard, the AHN classifier goes down 0.89% in accuracy and 0.46% in $F_{1}$-${\mathrm{score}}_{\mu }$. These comparisons give some insights about the robustness of the AHN classifier in contrast to the other two methods that were ranked in first place in any of the complete or reduced datasets.

### 6.2. Comparative Analysis on Supervised Model Performance under Noisy Data

A third experiment was conducted in order to measure the noise tolerance of the selected supervised classifiers. In this case, three noisy datasets (7%, 15% and 30% randomly corrupted) were used (see Section 5). Table 8, Table 9 and Table 10 show the overall results, sorted in descending order by accuracy, of this experiment.

In 7% noisy data, the AHN classifier ranks over the mean accuracy, and it is positioned in the first quartile of the evaluated classifiers. The proposed classifier is placed close to random forest (first place), stochastic gradient boosting (second place), rule-based (fourth place) and decision tree (fifth place) -based classifiers. In terms of the accuracy, the random forest-based classifier is 1.31% over the AHN classifier; while it is 0.71% over the AHN classifier in terms of $F_{1}$-${\mathrm{score}}_{\mu }$.

In 15% and 30% noisy data (Table 9 and Table 10), the AHN classifier also ranks over the mean accuracy, and it is positioned in the first quartile of the evaluated classifiers. In both experiments, the AHN classifier is very close to naive Bayes, *k*-nearest neighbors, SVM with radial basis function kernel and stochastic gradient boosting. In the 15% noisy dataset, the AHN classifier is ranked at the top of the table; while in the 30% noisy data, it is ranked 0.14% under the naive Bayes-based classifier.

### 6.3. Comparative Analysis on the Majority Voting Across Windows-Based Strategy

As already mentioned in Section 5, a majority voting across windows-based approach was also conducted to validate the performance of the AHN classifier in a simulated data flow that can be found in a real HAR system.

At first, we extracted the first 30 s of each activity carried out by each of the subjects (see Table 5), and we validated that our AHN classifier, as well as the other supervised models are able to classify human activities correctly. Table 11 reports the performance results of all methods, sorted in descending order by accuracy. In contrast with Table 6, it can be seen that the AHN classifier is stable in both circumstances with small (0.9829 in accuracy) and large (0.9845 in accuracy) testing sets. Furthermore, the other top methods (i.e., random forest, rule-based classifier, SVM, decision tree and stochastic gradient boosting) are consistent in both experiments. In addition, Table 12 shows the confusion matrix of the AHN classifier.

**Figure 6:**
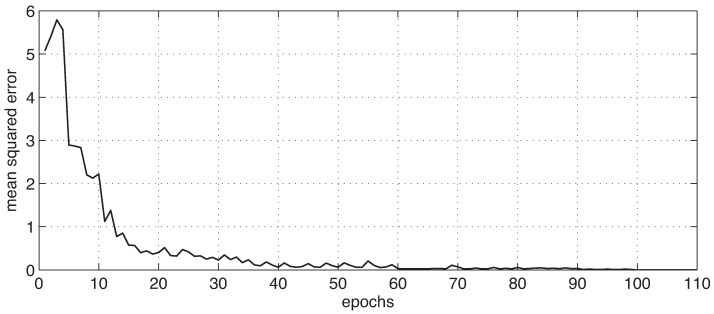
Learning curve of the AHN classifier for the windowing-based approach.

Then, a fixed window of 2.5 s was applied to the sequential data, and a majority voting strategy was computed within the window. The results of the AHN classifier, as well as the other fourteen methods are reported in Table 13, sorted in descending order by accuracy. Notice that the AHN classifier, as well as rule-based classifier, decision trees, random forest, stochastic gradient boosting and *k*-nearest neighbors have 100% accuracy. In particular, the confusion matrix of the AHN classifier is presented in Table 14. The values of this matrix correspond to the number of windows for each activity performed by the related subjects. In contrast with the confusion matrix of Table 12, the majority voting across windows-based approach improves the performance of the sample-based experiment. This can be explained because the latter has less false positive than true positive values for each activity. To this end, an overall perspective of the learning performance in the proposed classifier can be seen in Figure 6, which shows the learning curve of the AHN classifier for this experiment.

### 6.4. Discussion

From the first two experiments, the artificial hydrocarbon networks-based classifier showed good performance in terms of accuracy and $F_{1}$-${\mathrm{score}}_{\mu }$ in comparison with the other 14 supervised methods of classification. In that sense, the AHN classifier can achieve physical activity monitoring tasks.

Besides, Table 15, Table 16 and Table 17 show the confusion matrices of the AHN classifier using the 7%, 15% and 30% noisy datasets, respectively. As shown, the confusion matrices present a few mistaken classifications, most of them close to the diagonal. This behavior can be explained by the nature of the method. For instance, the nature of artificial hydrocarbon networks is mainly for regression tasks; then, classification problems are converted into a regression problem using numeric labels as data values for approximation. In that sense, similar numeric labels are the cause of misclassification. To this end, this misclassification behavior is completely related to the nature of the method and not in terms of the nature of physical activities.

On the other hand, large values in the confusion matrix are also analyzed. For instance, ascending stairs, cycling and walking are confused with Nordic walking; also, computer work is confused with watching TV. The human performances of these activities are closely related; thus, the performance of the AHN classifier is related to the nature of the physical activity. To this end, notice that confusion matrices correspond to the AHN classifier performance when data from sensors are corrupted, and as a result, it is more difficult to handle physical activity monitoring for the methods. From Table 8, Table 9 and Table 10, it is shown that the AHN classifier has a suitable performance in contrast with the other methods.

From the above experimental results, all methods have advantages and weaknesses. In that sense, the overall performance of the supervised classifiers is also inspected. For instance, Table 18 shows the overall performance of the classifiers in terms of the accuracy metric, and Table 19 summarizes the overall results in terms of the $F_{1}$-${\mathrm{score}}_{\mu }$. The first three experiments are concentrated in these tables. In order to preserve a more confident analysis, results from the 7% noisy dataset are only considered here. The mean ($\hat{x}$) and the standard deviation (*σ*) of both metrics were computed. The tables are sorted in descending order by the mean values of the metric, concluding that the artificial hydrocarbon networks-based classifier is ranked in second position in both accuracy and $F_{1}$-${\mathrm{score}}_{\mu }$ metrics.

**Figure 7:**
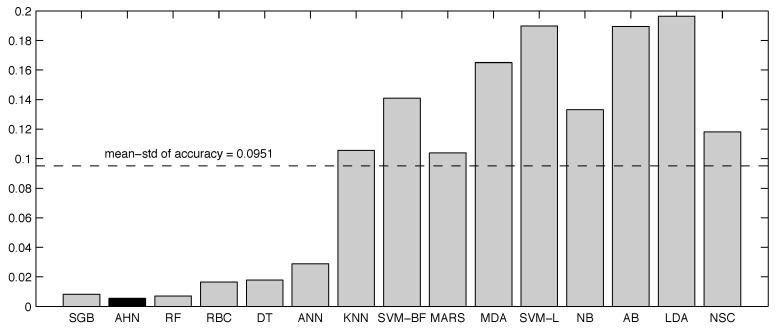
Overall variability of supervised classifiers present in the experiments with respect to the accuracy metric.

Since the accuracy measures the overall classification performance (Table 18), the AHN classifier is very competitive for physical activity monitoring ($\hat{x}=0.9756$) because the method is close to the best stochastic gradient boosting ranked method ($\hat{x}=0.9782$), representing a relative gap of 0.27%. In addition, the AHN classifier does not only performed well in monitoring, it also shows the smallest standard deviation (σ=0.0055) in comparison with the other methods, proving that the AHN classifier is very robust instead of different datasets (complete, reduced and noisy), as shown in Figure 7.

**Figure 8:**
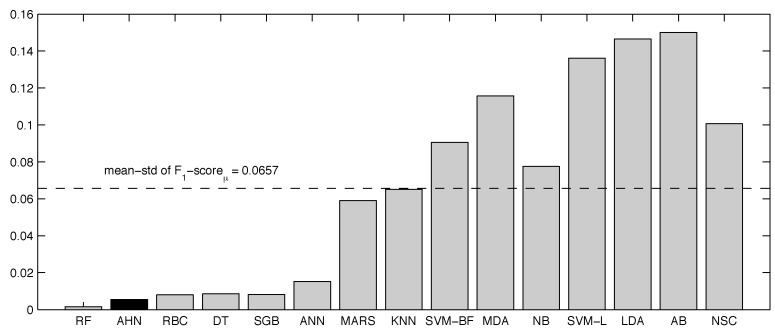
Overall variability of supervised classifiers present in the experiments with respect to the $F_{1}$-${\mathrm{score}}_{\mu }$ metric.

The same analysis can be done using the information from Table 19 in which the $F_{1}$-${\mathrm{score}}_{\mu }$ is compared. Since the $F_{1}$-${\mathrm{score}}_{\mu }$ measures the tradeoff between sensitivity and precision evaluations in unbalanced classes, then the AHN classifier is also suitable for physical activity monitoring represented by the $\hat{x}=0.9871$. This mean value is close to the best random forest ranked method, which obtained $\hat{x}=0.9895$, representing a relative gap of 0.24%. Using the $F_{1}$-${\mathrm{score}}_{\mu }$, the AHN classifier also showed suitable robustness to different datasets (complete, reduced and noisy), obtaining σ=0.0029, which ranks it in the second position below the random forest-based classifier, as depicted in Figure 8.

To this end, the AHN classifier is positioned close to the following classifiers in terms of monitoring task performance and noise tolerance (see Table 18 and Table 19) and robustness (see Figure 7 and Figure 8): Stochastic gradient boosting, random forest, rule-based classifier, decision trees and artificial neural networks.

A closer look at the results over the noisy datasets is summarized in Table 20. The mean and the standard deviation of accuracy and $F_{1}$-${\mathrm{score}}_{\mu }$ were calculated. As shown, the AHN classifier is ranked at the top of the table with 93.43% of accuracy and 96.97% of $F_{1}$-${\mathrm{score}}_{\mu }$ on average. In terms of standard deviation, the AHN classifier is the second best classifier in accuracy over the nearest shrunken centroids; and it is the best classifier in $F_{1}$-${\mathrm{score}}_{\mu }$. The above results conclude that the AHN classifier is tolerant to different ratios of noise in raw data sensors.

On the other hand, the above benchmark is closely related to the literature. An overall look into Table 18 and Table 19 shows that boosting and bagging methods (e.g., stochastic gradient boosting, AdaBoost and random forest) are positioned over discriminant analysis methods (e.g., linear and mixture), and those are over instance-based classifiers (e.g., *k*-nearest neighbors and nearest shrunken centroids), as noted in [25]. Furthermore, artificial neural networks are placed over discriminant analysis and instance-based methods, as suggested in [25]. In terms of noise tolerance, instance-based classifiers are easily altered by exclusion of single noisy data, as mentioned in [8]; this can be explained by the low positions of these methods observed in the experimental results. Additionally, decision trees obtained good performance in the benchmark (Table 8), which is correlated with the tolerance characteristic detected in [8], which assumes that decision trees trained with noisy data are more tolerant than when the method is trained with filtered data and then test data are corrupted with noise. With respect to support vector machines, the methods occupied in this benchmark obtained between medium to poor performance (see Table 8), which can be explained, since SVMs are easily altered by the exclusion of noisy data, as suggested in [8].

In fact, the above results were computed with raw sensor signals as features in order to minimize the influence of the feature extraction typically done in HAR. Hence, the accuracy in several methods is ranked high. Other factors that influence the high levels of accuracy are the cross-validation process and the selection of the best model based on the latter. In contrast to the single-based approach, a fourth experiment was conducted using a majority voting across windows-based approach. As noted, the proposed AHN classifier is improved in terms of accuracy (100%), since calculating a majority voting value per window increases the probability to predict activities well, as expected [32]. In addition, other methods can also reach that accuracy in the same way.

To this end, Table 21 summarizes the training time (measured in seconds) that classifiers take to build and train a model and the testing time (measured in milliseconds) that they take to compute a classification of one sample. As shown, the AHN classifier has the longest training times in both the complete (72.61 s) and the reduced (61.53 s) datasets; while it is the third worst classifier in terms of testing times in both the complete (1.71 ms) and the reduced (0.92 ms) datasets.

Finally, from the comparative study of the three experiments run in this benchmark, the majority voting across windows strategy and comparing the results obtained with the literature, it is evident that artificial hydrocarbon networks-based classifiers are: (i) suitable for physical activity monitoring; (ii) noise tolerant of corrupted data sensors; (iii) robust in terms of different issues for data sensors; and (iv) useful for simulated data flow classification; proving that AHN classifiers are suitable in the field of human activity recognition.

## 7. Conclusions and Future Work

Automated human activity recognition is a challenging task. Particularly in sensor-based approaches, these present several drawbacks, such as: the intensive periods of time for wearing sensors, typical battery issues and the presence of noise in data due to sensor errors or noisy environments. Thus, robust machine learning techniques are required in human activity recognition.

In that sense, this paper presents a novel supervised machine learning method called artificial hydrocarbon networks for human activity recognition. In fact, experimental results over a public physical activity monitoring raw dataset proved that the artificial hydrocarbon networks-based classifier is suitable for human activity recognition when compared to the other fourteen well-known supervised classifiers. In particular, the overall classification performance was measured in terms of accuracy ($\hat{x}=0.9756$) and micro-averaging $F_{1}$-${\mathrm{score}}_{\mu }$ ($\hat{x}=0.9871$), while robustness was analyzed in terms of the standard deviation of accuracy (σ=0.0055) and micro-averaging $F_{1}$-${\mathrm{score}}_{\mu }$ (σ=0.0029) over three different experiments, concluding that the AHN classifier is robust for different data (complete, reduced and noisy) profiles. To this end, experimental results in noisy data also confirm that the AHN classifier is noise tolerant of corrupted raw data sensors (i.e., 7%, 15% and 30% noise level), achieving 93.43% in accuracy and 96.97% in $F_{1}$-${\mathrm{score}}_{\mu }$. Moreover, when using a majority voting across windows-based approach, the AHN classifier is able to provide an accuracy of 100%, validating that it is useful for simulated data flow classification.

For future work, we must address two important challenges in order to prove that our AHN classifier is very well suited for human activity recognition. One important challenge for an activity recognition classifier is to determine if it is sufficiently flexible to deal with inter-person and intra-person differences in the activities’ performance. People can perform the same activity differently if they are in various times and situations (e.g., day or night, energetic or tired, etc.). Similarly, there is great variability in the performance of an activity depending on the person characteristics, such as age, weight, gender, health conditions, etc. [9]. The second challenge is to determine if AHN is capable of finding the most informative and discriminative features with the goal of developing a real-time HAR system to classify as many activities as possible with good performance. To this end, we will also revise the artificial hydrocarbon networks algorithm in order to improve the training time and make it more competitive with respect to the other methods.

## Figures and Tables

**Table 1:** Framework of artificial organic networks.

Framework Level	Description
implementation	*training* and *inference*
mathematical model	*structure* and *functionality*
chemical heuristic rules	*first molecules, then compounds, lastly mixtures*
interactions	*bonds in atoms and molecules, relations in compounds*
types of components	*atoms, molecules, compounds, mixtures*

**Table 2:** Physical activities identified in this case study, adapted from [13].

No.	Performed Activities	Activity Description
1	Lying	This movement is lying flat, slightly changing position or stretching a little bit.
2	Sitting	Refers to sitting in a chair in any posture. It also includes more comfortable positions as leaning or crossing your legs.
3	Standing	This position includes the natural movements of a person who is standing, swaying slightly, gesturing or talking.
4	Walking	This activity is a stroll down the street at a moderate speed of approximately 5 km/h.
5	Running	The people who made this activity ran at a moderate speed; taking into account non-high level athletes.
6	Cycling	A bicycle was used for this movement, and people pedaled as on a quiet ride. An activity requiring great effort was not requested.
7	Nordic walking	For this activity, it was required that persons that were inexperienced walked on asphalt using pads.
8	Watching TV	This position includes the typical movements of someone who is watching TV and changes the channel, lying on one side or stretching his or her legs.
9	Computer work	The typical movements of someone who works with a computer: mouse movement, movement of neck, etc.
10	Car driving	All movements necessary to move from the office to the house for testing sensors.
11	Ascending stairs	During this activity, the necessary movements up to a distance of five floors were recorded; from the ground floor to the fifth floor.
12	Descending stairs	This movement is the opposite of the former. Instead of climbing the stairs, the activity of descending them was recorded.
13	Vacuum cleaning	Refers to all of the activities necessary to clean a floor of the office. It also includes moving objects, such as rugs, chairs and wardrobes.
14	Ironing	It covers the necessary movements to iron a shirt or a t-shirt.
15	Folding laundry	It consists of folding clothes, such as shirts, pants and socks.
16	House cleaning	These are the movements that a person makes while cleaning a house; such as moving chairs to clean the floor, throwing things away, bending over to pick up something, etc.
17	Playing soccer	In this activity, individuals are negotiating, running the ball, shooting a goal or trying to stop the ball from the goal.
18	Rope jumping	There are people who prefer to jump with both feet together, and there are others who prefer to move one foot first and then the other.

**Table 3:** Configuration parameters of supervised models employed with the caret package in R.

No.	Method Name	Parameters	*Case 1* & *Case 3*	*Case 2*	Configurations
1	AdaBoost	size, decay, bag	(150, 3, 3)	(150, 3, 3)	27
2	Artificial Hydrocarbon Networks	molecules, eta, epsilon	(18, 0.1, 0.0001)	(18, 0.1, 0.0001)	1
3	C4.5 Decision Trees	c	(0.25)	(0.25)	1
4	*k*-Nearest Neighbors	kmax, distance, kernel	(9, 2, 1)	(9, 2, 1)	3
5	Linear Discriminant Analysis	–	–	–	1
6	Mixtures Discriminant Analysis	subclasses	(4)	(4)	3
7	Multivariate Adaptive Regression Splines	degree	(1)	(1)	1
8	Naive Bayes	fl, use_kernel	(0, true)	(0, true)	2
9	Nearest Shrunken Centroids	threshold	(2.512)	(1.363)	3
10	Artificial Neural Networks	size, decay	(5, 0)	(5, 0)	9
11	Random Forest	mtry	(26)	(6)	3
12	Rule-Based Classifier	threshold, pruned	(0.25, true)	(0.25, true)	1
13	Stochastic Gradient Boosting	n.trees, depth, shrinkage	(150, 3, 0.1)	(150, 2, 0.1)	9
14	SVM with Linear Kernel	c	(1)	(1)	1
15	SVM with Radial Basis Function Kernel	sigma, c	(0.0179, 1)	(0.0748, 1)	3

**Table 4:** Retained features using the recursive feature elimination (RFE) technique.

Sensor	Features Selected	Feature Number
*hand*	temperature	4
*chest*	temperature	21
	z-axis 3D-accelerometer 16 g	24
	z-axis 3D-accelerometer 6 g	27
	y-axis 3D-magnetometer	32
	z-axis 3D-magnetometer	33
*ankle*	temperature	38
	x-axis 3D-magnetometer	48
	z-axis 3D-magnetometer	50
	first orientation	51

**Table 5:** Summary of activities performed by each subject for at least 30 s.

Activities	Sub 1	Sub 2	Sub 3	Sub 4	Sub 5	Sub 6	Sub 7	Sub 8	Sub 9	Total
Lying	-	-	-	-	-	-	-	-		8
Sitting	-	-	-	-	-	-	-	-		8
Standing	-	-	-	-	-	-	-	-		8
Walking	-	-	-	-	-	-	-	-		8
Running	-	-			-	-		-		5
Cycling	-	-		-	-	-	-	-		7
Nordic walking	-	-		-	-	-	-	-		7
Watching TV	-									1
Computer work					-	-		-	-	4
Car driving	-									1
Ascending stairs	-	-	-	-	-	-	-	-		8
Descending stairs	-	-	-	-	-	-	-	-		8
Vacuum cleaning	-	-	-	-	-	-	-	-		8
Ironing	-	-	-	-	-	-	-	-		8
Folding laundry	-					-		-	-	4
House cleaning	-				-	-		-	-	5
**Playing soccer**								-	-	2
Rope jumping	-	-			-			-	-	5

**Table 6:** Comparison of the supervised classifiers using the complete dataset of the case study.

No.	Method Name	Accuracy	Sensitivity${}_{\mu }$	Precision${}_{\mu }$	$F_{1}$-Score${}_{\mu }$	Sensitivity${}_{M}$	Precision${}_{M}$	$F_{1}$-Score${}_{M}$
1	C4.5 Decision Trees	0.9880	0.9994	0.9893	0.9943	0.9886	0.9885	0.9938
2	Rule-Based Classifier	0.9876	0.9993	0.9890	0.9942	0.9883	0.9882	0.9937
**3**	**Artificial Hydrocarbon Networks**	**0.9829**	**0.9829**	**0.9831**	**0.9910**	**0.9830**	**0.9832**	**0.9910**
4	SVM with Linear Kernel	0.9827	0.9989	0.9828	0.9908	0.9833	0.9834	0.9911
5	SVM with Radial Basis Function Kernel	0.9745	0.9985	0.9752	0.9867	0.9746	0.9747	0.9865
6	Random Forest	0.9727	0.9986	0.9819	0.9902	0.9743	0.9797	0.9890
7	Stochastic Gradient Boosting	0.9725	0.9725	0.9805	0.9893	0.9740	0.9815	0.9899
8	k-Nearest Neighbors	0.9718	0.9984	0.9724	0.9852	0.9711	0.9717	0.9849
9	Mixture Discriminant Analysis	0.9714	0.9982	0.9717	0.9848	0.9724	0.9729	0.9854
10	AdaBoost	0.9710	0.9982	0.9774	0.9877	0.9726	0.9780	0.9880
11	Multivariate Adaptive Regression Splines	0.9553	0.9974	0.9621	0.9794	0.9572	0.9609	0.9788
12	Linear Discriminant Analysis	0.9382	0.9962	0.9398	0.9672	0.9399	0.9418	0.9683
13	Naive Bayes	0.9327	0.9962	0.9459	0.9704	0.9350	0.9438	0.9692
14	Artificial Neural Networks	0.8976	0.9939	0.9019	0.9457	0.8984	0.9020	0.9458
15	Nearest Shrunken Centroids	0.7031	0.9820	0.7006	0.8178	0.7073	0.7063	0.8218
	**Average**	**0.9468**	**0.9941**	**0.9502**	**0.9716**	**0.9480**	**0.9504**	**0.9718**

**Table 7:** Comparison of the supervised classifiers using the reduced dataset of the case study.

No.	Method Name	Accuracy	Sensitivity${}_{\mu }$	Precision${}_{\mu }$	$F_{1}$-Score${}_{\mu }$	Sensitivity${}_{M}$	Precision${}_{M}$	$F_{1}$-Score${}_{M}$
1	Stochastic Gradient Boosting	0.9898	0.9898	0.9907	0.9950	0.9900	0.9900	0.9947
**2**	**Artificial Hydrocarbon Networks**	**0.9741**	**0.9741**	**0.9746**	**0.9864**	**0.9744**	**0.9741**	**0.9861**
3	AdaBoost	0.9657	0.9979	0.9674	0.9824	0.9675	0.9686	0.9831
4	Random Forest	0.9655	0.9982	0.9768	0.9874	0.9673	0.9744	0.9861
5	Rule-Based Classifier	0.9604	0.9979	0.9716	0.9846	0.9617	0.9693	0.9833
6	C4.5 Decision Trees	0.9571	0.9976	0.9647	0.9809	0.9586	0.9629	0.9799
7	k-Nearest Neighbors	0.7222	0.9834	0.7217	0.8325	0.7291	0.7255	0.8351
8	Multivariate Adaptive Regression Splines	0.7061	0.9825	0.7357	0.8413	0.7159	0.7392	0.8437
9	SVM with Radial Basis Function Kernel	0.6549	0.9791	0.6526	0.7832	0.6619	0.6601	0.7887
10	Naive Bayes	0.6069	0.9758	0.6506	0.7807	0.6125	0.6615	0.7888
11	Mixture Discriminant Analysis	0.5853	0.9750	0.5684	0.7181	0.5929	0.5746	0.7232
12	SVM with Linear Kernel	0.5386	0.9720	0.5196	0.6772	0.5487	0.5296	0.6858
13	Artificial Neural Networks	0.5092	0.9703	0.4631	0.6270	0.5190	0.4716	0.6349
14	Linear Discriminant Analysis	0.4792	0.9683	0.4676	0.6307	0.4853	0.4773	0.6396
15	Nearest Shrunken Centroids	0.4216	0.9645	0.4131	0.5784	0.4191	0.4209	0.5863
	**Average**	**0.7358**	**0.9818**	**0.7359**	**0.8257**	**0.7403**	**0.7400**	**0.8293**

**Table 8:** Comparison of the supervised classifiers using the 7% noisy dataset of the case study.

No.	Method Name	Accuracy	Sensitivity${}_{\mu }$	Precision${}_{\mu }$	$F_{1}$-Score${}_{\mu }$	Sensitivity${}_{M}$	Precision${}_{M}$	$F_{1}$-Score${}_{M}$
1	Random Forest	0.9825	0.9990	0.9830	0.9909	0.9833	0.9830	0.9909
2	Stochastic Gradient Boosting	0.9722	0.9722	0.9754	0.9868	0.9733	0.9747	0.9864
**3**	**Artificial Hydrocarbon Networks**	**0.9696**	**0.9696**	**0.9700**	**0.9839**	**0.9691**	**0.9696**	**0.9837**
4	Rule-Based Classifier	0.9484	0.9970	0.9532	0.9747	0.9497	0.9521	0.9740
5	C4.5 Decision Trees	0.9459	0.9969	0.9519	0.9739	0.9478	0.9507	0.9732
6	SVM with Radial Basis Function Kernel	0.9276	0.9957	0.9281	0.9607	0.9278	0.9285	0.9609
7	AdaBoost	0.9151	0.9949	0.9242	0.9582	0.9181	0.9256	0.9590
8	*k*-Nearest Neighbors	0.9059	0.9944	0.9066	0.9484	0.9060	0.9075	0.9490
9	Mixture Discriminant Analysis	0.8816	0.9928	0.8830	0.9347	0.8831	0.8856	0.9362
10	SVM with Linear Kernel	0.8796	0.9928	0.8786	0.9322	0.8800	0.8792	0.9326
11	Multivariate Adaptive Regression Splines	0.8755	0.9924	0.9055	0.9469	0.8759	0.9083	0.9486
12	Linear Discriminant Analysis	0.8339	0.9899	0.8367	0.9069	0.8353	0.8398	0.9088
13	Artificial Neural Networks	0.7820	0.9870	0.7764	0.8691	0.7842	0.7782	0.8703
14	Naive Bayes	0.7769	0.9868	0.7957	0.8810	0.7811	0.7964	0.8815
15	Nearest Shrunken Centroids	0.6202	0.9771	0.6076	0.7493	0.6235	0.6125	0.7531
	**Average**	**0.8811**	**0.9892**	**0.8851**	**0.9332**	**0.8825**	**0.8861**	**0.9339**

**Table 9:** Comparison of the supervised classifiers using the 15% noisy dataset of the case study.

No.	Method Name	Accuracy	Sensitivity${}_{\mu }$	Precision${}_{\mu }$	$F_{1}$-Score${}_{\mu }$	Sensitivity${}_{M}$	Precision${}_{M}$	$F_{1}$-Score${}_{M}$
**1**	**Artificial Hydrocarbon Networks**	**0.9547**	**0.9547**	**0.9596**	**0.9781**	**0.9546**	**0.9592**	**0.9779**
2	*k*-Nearest Neighbors	0.9425	0.9425	0.9433	0.9692	0.9418	0.9433	0.9692
3	SVM with Radial Basis Function Kernel	0.9249	0.9249	0.9308	0.9621	0.9238	0.9314	0.9624
4	Naive Bayes	0.9182	0.9182	0.9343	0.9638	0.9209	0.9328	0.9630
5	Stochastic Gradient Boosting	0.8402	0.8402	0.8729	0.9278	0.8387	0.8767	0.9302
6	SVM with Linear Kernel	0.8400	0.8400	0.8550	0.9177	0.8394	0.8579	0.9195
7	Mixture Discriminant Analysis	0.7975	0.7975	0.8113	0.8909	0.7986	0.8148	0.8931
8	AdaBoost	0.7755	0.7755	0.8209	0.8960	0.7724	0.8252	0.8988
9	Random Forest	0.7498	0.7498	0.8263	0.8986	0.7477	0.8313	0.9017
10	Linear Discriminant Analysis	0.7478	0.7478	0.7668	0.8622	0.7489	0.7710	0.8650
11	Rule-Based Classifier	0.7414	0.7414	0.8039	0.8851	0.7370	0.8043	0.8855
12	C4.5 Decision Trees	0.7335	0.7335	0.7984	0.8815	0.7290	0.8014	0.8835
13	Multivariate Adaptive Regression Splines	0.6824	0.6824	0.7610	0.8569	0.6791	0.7684	0.8619
14	Nearest Shrunken Centroids	0.6761	0.6761	0.6820	0.8044	0.6809	0.6884	0.8090
15	Artificial Neural Networks	0.5214	0.5214	0.5339	0.6892	0.5253	0.5307	0.6865
	**Average**	**0.7897**	**0.7897**	**0.8200**	**0.8922**	**0.7892**	**0.8225**	**0.8938**

**Table 10:** Comparison of the supervised classifiers using the 30% noisy dataset of the case study.

No.	Method Name	Accuracy	Sensitivity${}_{\mu }$	Precision${}_{\mu }$	$F_{1}$-Score${}_{\mu }$	Sensitivity${}_{M}$	Precision${}_{M}$	$F_{1}$-score${}_{M}$
1	Naive Bayes	0.8798	0.8798	0.9090	0.9491	0.8816	0.9087	0.9489
**2**	**Artificial Hydrocarbon Networks**	**0.8786**	**0.8786**	**0.9052**	**0.9470**	**0.8793**	**0.9044**	**0.9466**
3	*k*-Nearest Neighbors	0.8608	0.8608	0.8681	0.9257	0.8604	0.8708	0.9273
4	SVM with Radial Basis Function Kernel	0.8043	0.8043	0.8349	0.9051	0.8024	0.8374	0.9067
5	Stochastic Gradient Boosting	0.6967	0.6967	0.7822	0.8705	0.6950	0.7905	0.8759
6	SVM with Linear Kernel	0.6747	0.6747	0.7365	0.8410	0.6750	0.7419	0.8448
7	Nearest Shrunken Centroids	0.6302	0.6302	0.6605	0.7883	0.6354	0.6675	0.7935
8	AdaBoost	0.6302	0.6302	0.7315	0.8367	0.6249	0.7392	0.8421
9	Mixture Discriminant Analysis	0.6098	0.6098	0.6525	0.7823	0.6116	0.6579	0.7863
10	Random Forest	0.5514	0.5514	0.7378	0.8390	0.5474	0.7463	0.8449
11	Rule-Based Classifier	0.5496	0.5496	0.6913	0.8082	0.5446	0.6926	0.8093
12	Linear Discriminant Analysis	0.5449	0.5449	0.5988	0.7412	0.5483	0.6050	0.7461
13	C4.5 Decision Trees	0.5308	0.5308	0.6693	0.7925	0.5255	0.6760	0.7976
14	Multivariate Adaptive Regression Splines	0.4688	0.4688	0.6439	0.7731	0.4634	0.6548	0.7814
15	Artificial Neural Networks	0.4559	0.4559	0.5271	0.6824	0.4563	0.5295	0.6845
	**Average**	**0.6511**	**0.6511**	**0.7299**	**0.8321**	**0.6501**	**0.7348**	**0.8357**

**Table 11:** Comparison of the supervised classifiers using the first 30 s of each activity carried out by all subjects.

No.	Method Name	Accuracy	Sensitivity${}_{\mu }$	Precision${}_{\mu }$	$F_{1}$-Score${}_{\mu }$	Sensitivity${}_{M}$	Precision${}_{M}$	$F_{1}$-Score${}_{M}$
1	Stochastic Gradient Boosting	0.9952	0.9952	0.9996	0.9974	0.9963	0.9962	0.9980
2	Random Forest	0.9951	0.9951	0.9996	0.9974	0.9963	0.9959	0.9978
3	Rule-Based Classifier	0.9866	0.9866	0.9990	0.9928	0.9894	0.9857	0.9924
4	**Artificial Hydrocarbon Networks**	**0.9845**	**0.9845**	**0.9991**	**0.9919**	**0.9844**	**0.9752**	**0.9870**
5	SVM with Radial Basis Function Kernel	0.9635	0.9635	0.9973	0.9804	0.9698	0.9662	0.9818
6	C4.5 Decision Trees	0.9630	0.9630	0.9973	0.9797	0.9709	0.9652	0.9812
7	Multivariate Adaptive Regression Splines	0.9574	0.9574	0.9966	0.9778	0.9669	0.9673	0.9822
8	AdaBoost	0.9474	0.9474	0.9959	0.9736	0.9498	0.9621	0.9792
9	SVM with Linear Kernel	0.9441	0.9441	0.9958	0.9690	0.9550	0.9508	0.9732
10	*k*-Nearest Neighbors	0.9141	0.9140	0.9942	0.9530	0.9183	0.9122	0.9518
11	Naive Bayes	0.9093	0.9093	0.9939	0.9518	0.9208	0.9150	0.9532
12	Artificial Neural Networks	0.8879	0.8879	0.9923	0.9388	0.9032	0.8909	0.9393
13	Mixture Discriminant Analysis	0.8868	0.8868	0.9922	0.9379	0.9026	0.8914	0.9396
14	Linear Discriminant Analysis	0.7748	0.7748	0.9847	0.8700	0.7941	0.7797	0.8711
15	Nearest Shrunken Centroids	0.6199	0.6199	0.9727	0.7350	0.6285	0.5972	0.7414
	**Average**	**0.9153**	**0.9153**	**0.9940**	**0.9498**	**0.9231**	**0.9167**	**0.9513**

**Table 12:** Confusion matrix of the AHN classifier using the first 30 s of each activity carried out by all subjects.

		Actual Values																	
		Lying	Sitting	Standing	Walking	Running	Cycling	Nordic walking	Watching TV	Computer Work	Car Driving	Ascending Stairs	Descending Stairs	Vacuum Cleaning	Ironing	Folding laundry	House Cleaning	Playing Soccer	Rope Jumping
	Lying	**23985**	19	1	22	30	7	13	3	0	6	0	10	0	0	0	0	3	0
	Sitting	13	**23567**	6	22	8	1	1	0	0	0	0	4	0	0	0	0	0	1
	Standing	0	20	**23542**	30	10	5	11	0	0	0	1	4	0	0	0	0	0	0
	Walking	2	109	48	**23567**	19	16	38	0	0	0	7	8	0	0	0	0	0	0
	Running	0	133	47	70	**14760**	14	78	0	0	0	7	11	1	0	0	0	1	0
	Cycling	0	47	75	85	19	**20616**	66	0	0	0	10	21	1	0	0	0	1	1
	Nordic walking	0	74	129	134	32	120	**20676**	0	65	3	43	41	14	2	0	7	3	1
	Watching TV	0	4	69	31	26	73	35	**2961**	35	10	27	29	17	2	0	19	1	1
	Computer work	0	4	18	17	21	51	21	33	**11790**	7	37	31	31	9	3	18	2	0
	Car driving	0	7	12	6	22	37	14	0	37	**2957**	49	50	41	20	1	27	4	2
	Ascending stairs	0	1	6	1	10	21	19	0	36	4	**23638**	55	66	42	9	11	6	2
Predicted values	Descending stairs	0	0	2	3	10	10	10	0	17	1	64	**23569**	72	72	12	33	8	8
	Vacuum cleaning	0	0	17	3	17	12	13	0	20	2	90	103	**23684**	190	70	90	30	40
	Ironing	0	7	26	0	4	0	0	0	0	0	7	16	30	**23592**	34	34	8	20
	Folding laundry	0	0	0	1	1	0	0	0	0	0	6	20	24	41	**11820**	27	15	15
	House cleaning	0	0	0	0	3	0	0	0	0	0	2	8	14	19	22	**14690**	11	14
	Playing soccer	0	0	0	0	4	0	0	0	0	0	6	12	5	9	28	32	**5883**	69
	Rope jumping	0	8	2	8	4	17	5	3	0	10	6	8	0	2	1	12	24	**14826**

**Table 13:** Comparison of the supervised classifiers using a majority voting across windows-based approach (2.5-s window size).

No.	Method Name	Accuracy	Sensitivity${}_{\mu }$	Precision${}_{\mu }$	$F_{1}$-Score${}_{\mu }$	Sensitivity${}_{M}$	Precision${}_{M}$	$F_{1}$-Score${}_{M}$
**1**	**Artificial Hydrocarbon Networks**	**1.0**	**1.0**	**1.0**	**1.0**	**1.0**	**1.0**	**1.0**
2	Rule-Based Classifier	1.0	1.0	1.0	1.0	1.0	1.0	1.0
3	C4.5 Decision Trees	1.0	1.0	1.0	1.0	1.0	1.0	1.0
4	Random Forest	1.0	1.0	1.0	1.0	1.0	1.0	1.0
5	Stochastic Gradient Boosting	1.0	1.0	1.0	1.0	1.0	1.0	1.0
6	*k*-Nearest Neighbors	1.0	1.0	1.0	1.0	1.0	1.0	1.0
7	SVM with Radial Basis Function Kernel	0.9841	0.9841	0.9846	0.9916	0.9873	0.9886	0.9938
8	SVM with Linear Kernel	0.9786	0.9786	0.9791	0.9887	0.9843	0.9821	0.9904
9	Multivariate Adaptive Regression Splines	0.9770	0.9770	0.9785	0.9882	0.9832	0.9844	0.9914
10	AdaBoost	0.9627	0.9627	0.9651	0.9808	0.9681	0.9732	0.9853
11	Naive Bayes	0.9571	0.9571	0.9604	0.9785	0.9651	0.9603	0.9785
12	Artificial Neural Networks	0.9460	0.9460	0.9478	0.9714	0.9570	0.9481	0.9718
13	Mixture Discriminant Analysis	0.9365	0.9365	0.9412	0.9677	0.9500	0.9421	0.9684
14	Linear Discriminant Analysis	0.8444	0.8444	0.8508	0.9148	0.8583	0.8528	0.9166
15	Nearest Shrunken Centroids	0.6857	0.6857	0.6593	0.7872	0.7018	0.6773	0.8014
	**Average**	**0.9515**	**0.9515**	**0.9511**	**0.9713**	**0.9570**	**0.9539**	**0.9732**

**Table 14:** Confusion matrix of the artificial hydrocarbon networks (AHN) classifier using a majority voting across windows-based approach (2.5-s window size).

		Actual Values																	
		Lying	Sitting	Standing	Walking	Running	Cycling	Nordic Walking	Watching TV	Computer Work	Car Driving	Ascending Stairs	Descending Stairs	Vacuum Cleaning	Ironing	Folding Laundry	House Cleaning	Playing Soccer	Rope Jumping
	Lying	**96**	0	0	0	0	0	0	0	0	0	0	0	0	0	0	0	0	0
	Sitting	0	**96**	0	0	0	0	0	0	0	0	0	0	0	0	0	0	0	0
	Standing	0	0	**96**	0	0	0	0	0	0	0	0	0	0	0	0	0	0	0
	Walking	0	0	0	**96**	0	0	0	0	0	0	0	0	0	0	0	0	0	0
	Running	0	0	0	0	**60**	0	0	0	0	0	0	0	0	0	0	0	0	0
	Cycling	0	0	0	0	0	**84**	0	0	0	0	0	0	0	0	0	0	0	0
	Nordic walking	0	0	0	0	0	0	**84**	0	0	0	0	0	0	0	0	0	0	0
	Watching TV	0	0	0	0	0	0	0	**12**	0	0	0	0	0	0	0	0	0	0
	Computer work	0	0	0	0	0	0	0	0	**48**	0	0	0	0	0	0	0	0	0
	Car driving	0	0	0	0	0	0	0	0	0	**12**	0	0	0	0	0	0	0	0
	Ascending stairs	0	0	0	0	0	0	0	0	0	0	**96**	0	0	0	0	0	0	0
Predicted values	Descending stairs	0	0	0	0	0	0	0	0	0	0	0	**96**	0	0	0	0	0	0
	Vacuum cleaning	0	0	0	0	0	0	0	0	0	0	0	0	**96**	0	0	0	0	0
	Ironing	0	0	0	0	0	0	0	0	0	0	0	0	0	**96**	0	0	0	0
	Folding laundry	0	0	0	0	0	0	0	0	0	0	0	0	0	0	**48**	0	0	0
	House cleaning	0	0	0	0	0	0	0	0	0	0	0	0	0	0	0	**60**	0	0
	Playing soccer	0	0	0	0	0	0	0	0	0	0	0	0	0	0	0	0	**24**	0
	Rope jumping	0	0	0	0	0	0	0	0	0	0	0	0	0	0	0	0	0	**60**

**Table 15:** Confusion matrix of the AHN classifier using the 7% noisy dataset.

		Actual Values																	
		Lying	Sitting	Standing	Walking	Running	Cycling	Nordic Walking	Watching TV	Computer Work	Car Driving	Ascending Stairs	Descending Stairs	Vacuum Cleaning	Ironing	Folding Laundry	House Cleaning	Playing Soccer	Rope Jumping
	Lying	**297**	3	0	1	0	0	0	0	0	0	0	0	0	0	0	0	1	0
	Sitting	0	**284**	1	0	2	0	0	0	0	0	0	0	0	0	0	0	0	0
	Standing	0	3	**291**	1	1	0	0	0	0	0	0	0	0	0	0	0	0	0
	Walking	1	3	0	**292**	2	0	1	1	0	0	0	0	0	1	0	0	0	0
	Running	0	3	1	0	**291**	1	1	0	0	0	0	0	1	0	0	0	0	0
	Cycling	0	1	0	2	1	**294**	1	1	0	1	0	0	1	0	0	0	0	0
	Nordic walking	0	2	3	3	2	4	**291**	3	3	1	5	1	1	0	0	0	0	0
	Watching TV	0	0	0	0	0	1	4	**241**	4	2	0	1	0	0	0	1	0	0
	Computer work	0	1	2	1	1	0	0	1	**241**	3	1	2	0	0	1	1	0	0
	Car driving	0	0	0	0	0	0	1	2	2	**240**	0	1	1	0	1	0	0	0
	Ascending stairs	0	0	0	0	0	0	1	0	0	1	**291**	1	0	2	1	0	0	0
Predicted values	Descending stairs	0	0	2	0	0	0	0	0	0	1	1	**292**	1	1	1	1	1	0
	Vacuum cleaning	1	0	0	0	0	0	0	0	0	1	2	1	**293**	2	1	3	2	0
	Ironing	0	0	0	0	0	0	0	0	0	0	0	0	0	**293**	0	0	1	1
	Folding laundry	0	0	0	0	0	0	0	0	0	0	0	0	1	0	**241**	2	0	0
	House cleaning	0	0	0	0	0	0	0	0	0	0	0	1	0	1	2	**239**	4	1
	Playing soccer	1	0	0	0	0	0	0	0	0	0	0	0	1	0	1	3	**239**	3
	Rope jumping	0	0	0	0	0	0	0	1	0	0	0	0	0	0	1	0	2	**295**

**Table 16:** Confusion matrix of the AHN classifier using the 15% noisy dataset.

		Actual Values																	
		Lying	Sitting	Standing	Walking	Running	Cycling	Nordic Walking	Watching TV	Computer Work	Car Driving	Ascending Stairs	Descending Stairs	Vacuum Cleaning	Ironing	Folding Laundry	House Cleaning	Playing Soccer	Rope Jumping
	Lying	**288**	0	0	0	0	0	0	0	0	0	0	0	0	0	0	0	1	0
	Sitting	0	**286**	0	0	0	0	0	0	0	0	0	0	0	0	0	0	0	0
	Standing	0	0	**283**	0	0	0	0	0	0	0	0	0	0	0	0	0	0	0
	Walking	0	0	0	**285**	0	0	0	0	0	0	0	0	0	0	0	0	0	0
	Running	0	0	0	0	**284**	1	0	0	0	0	0	0	0	0	0	1	0	0
	Cycling	0	0	2	1	1	**285**	1	8	0	0	1	2	0	2	0	2	0	0
	Nordic walking	6	12	4	9	6	7	**294**	6	3	1	6	11	5	12	2	5	0	0
	Watching TV	0	0	1	1	2	3	0	**236**	6	0	2	0	2	0	3	1	0	1
	Computer work	0	1	3	1	2	0	2	0	**238**	4	1	1	0	1	3	1	0	0
	Car driving	4	0	2	3	0	2	3	0	2	**242**	3	0	2	1	2	2	0	0
	Ascending stairs	1	1	3	0	3	1	0	0	1	1	**287**	3	2	0	2	2	0	0
Predicted values	Descending stairs	0	0	2	0	2	1	0	0	0	2	0	**283**	0	0	2	1	0	0
	Vacuum cleaning	1	0	0	0	0	0	0	0	0	0	0	0	**289**	0	0	0	1	0
	Ironing	0	0	0	0	0	0	0	0	0	0	0	0	0	**284**	0	0	1	1
	Folding laundry	0	0	0	0	0	0	0	0	0	0	0	0	0	0	**236**	0	1	0
	House cleaning	0	0	0	0	0	0	0	0	0	0	0	0	0	0	0	**235**	0	0
	Playing soccer	0	0	0	0	0	0	0	0	0	0	0	0	0	0	0	0	**241**	5
	Rope jumping	0	0	0	0	0	0	0	0	0	0	0	0	0	0	0	0	5	**293**

**Table 17:** Confusion matrix of the AHN classifier using the 30% noisy dataset.

		Actual Values																	
		Lying	Sitting	Standing	Walking	Running	Cycling	Nordic Walking	Watching TV	Computer Work	Car Driving	Ascending Stairs	Descending Stairs	Vacuum Cleaning	Ironing	Folding Laundry	House Cleaning	Playing Soccer	Rope Jumping
	Lying	**263**	0	0	0	0	0	0	0	0	0	0	0	0	0	0	0	2	0
	Sitting	0	**268**	0	0	0	0	0	0	0	0	0	0	0	0	0	0	0	0
	Standing	0	0	**244**	0	0	0	0	0	0	0	0	0	0	0	0	0	0	0
	Walking	0	0	0	**253**	0	0	0	0	0	0	0	0	0	0	0	0	0	0
	Running	0	2	0	0	**254**	0	0	0	2	0	0	1	0	0	1	0	0	0
	Cycling	3	4	1	1	1	**253**	2	9	1	3	2	0	1	5	3	0	1	0
	Nordic walking	17	22	20	26	19	16	**278**	20	6	3	16	17	19	31	8	10	1	0
	Watching TV	1	0	13	1	5	5	8	**221**	2	0	2	2	5	4	2	3	0	0
	Computer work	1	0	4	2	2	5	5	0	**221**	4	3	2	9	0	5	3	0	0
	Car driving	7	2	3	10	5	6	3	0	9	**231**	4	6	4	2	9	2	1	1
	Ascending stairs	5	1	13	5	9	10	4	0	4	9	**269**	5	7	1	5	6	1	0
Predicted values	Descending stairs	2	1	2	1	4	3	0	0	3	0	1	**265**	1	0	4	1	1	0
	Vacuum cleaning	1	0	0	1	1	2	0	0	2	0	3	2	**254**	1	1	0	7	1
	Ironing	0	0	0	0	0	0	0	0	0	0	0	0	0	**256**	0	0	0	0
	Folding laundry	0	0	0	0	0	0	0	0	0	0	0	0	0	0	**212**	0	1	2
	House cleaning	0	0	0	0	0	0	0	0	0	0	0	0	0	0	0	**225**	2	1
	Playing soccer	0	0	0	0	0	0	0	0	0	0	0	0	0	0	0	0	**225**	6
	Rope jumping	0	0	0	0	0	0	0	0	0	0	0	0	0	0	0	0	8	**289**

**Table 18:** Overall performance of supervised classifiers during the experiments with respect to the $\hat{x}$ accuracy metric.

No.	Method Name	Complete Dataset	7% Noisy Dataset	Reduced Dataset	$\hat{x}$ Accuracy	*σ* Accuracy
1	Stochastic Gradient Boosting	0.9725	0.9722	0.9898	0.9782	0.0082
**2**	**Artificial Hydrocarbon Networks**	**0.9829**	**0.9696**	**0.9741**	**0.9756**	**0.0055**
3	Random Forest	0.9727	0.9825	0.9655	0.9736	0.0070
4	Rule-Based Classifier	0.9876	0.9484	0.9604	0.9655	0.0164
5	C4.5 Decision Trees	0.9880	0.9459	0.9571	0.9637	0.0178
6	Artificial Neural Networks	0.8976	0.9151	0.9657	0.9261	0.0289
7	*k*-Nearest Neighbors	0.9718	0.9059	0.7222	0.8666	0.1056
8	SVM with Radial Basis Function Kernel	0.9745	0.9276	0.6549	0.8524	0.1409
9	Multivariate Adaptive Regression Splines	0.9553	0.8755	0.7061	0.8456	0.1039
10	Mixture Discriminant Analysis	0.9714	0.8816	0.5853	0.8127	0.1650
11	SVM with Linear Kernel	0.9827	0.8796	0.5386	0.8003	0.1898
12	Naive Bayes	0.9327	0.7769	0.6069	0.7722	0.1331
13	AdaBoost	0.9710	0.7820	0.5092	0.7541	0.1895
14	Linear Discriminant Analysis	0.9382	0.8339	0.4792	0.7505	0.1965
15	Nearest Shrunken Centroids	0.7031	0.6202	0.4216	0.5816	0.1181
	**Average**	**0.9468**	**0.8811**	**0.7358**	**0.8546**	**0.0951**

**Table 19:** Overall performance of supervised classifiers during the experiments with respect to the $\hat{x}$ $F_{1}$-${\mathrm{score}}_{\mu }$ metric.

No.	Method Name	Complete Dataset	7% Noisy Dataset	Reduced Dataset	$\hat{x}$ $F_{1}$-Score${}_{\mu }$	*σ* $F_{1}$-Score${}_{\mu }$
1	Random Forest	0.9902	0.9909	0.9874	0.9895	0.0015
**2**	**Artificial Hydrocarbon Networks**	**0.9910**	**0.9839**	**0.9864**	**0.9871**	**0.0029**
3	Rule-Based Classifier	0.9942	0.9747	0.9846	0.9845	0.0080
4	C4.5 Decision Trees	0.9943	0.9739	0.9809	0.9830	0.0085
5	Stochastic Gradient Boosting	0.9725	0.9722	0.9898	0.9782	0.0082
6	Artificial Neural Networks	0.9457	0.9582	0.9824	0.9621	0.0152
7	Multivariate Adaptive Regression Splines	0.9794	0.9469	0.8413	0.9226	0.0590
8	*k*-Nearest Neighbors	0.9852	0.9484	0.8325	0.9220	0.0651
9	SVM with Radial Basis Function Kernel	0.9867	0.9607	0.7832	0.9102	0.0905
10	Mixture Discriminant Analysis	0.9848	0.9347	0.7181	0.8792	0.1157
11	Naive Bayes	0.9704	0.8810	0.7807	0.8774	0.0775
12	SVM with Linear Kernel	0.9908	0.9322	0.6772	0.8667	0.1362
13	Linear Discriminant Analysis	0.9672	0.9069	0.6307	0.8349	0.1465
14	AdaBoost	0.9877	0.8691	0.6270	0.8279	0.1501
15	Nearest Shrunken Centroids	0.8178	0.7493	0.5784	0.7152	0.1006
	**Average**	**0.9705**	**0.9322**	**0.8254**	**0.9094**	**0.0657**

**Table 20:** Overall performance of supervised classifiers during the experiments with 7%, 15% and 30% noisy datasets.

No.	Method Name	$\hat{x}$ Accuracy	*σ* Accuracy	7% (acc)	15% (acc)	30% (acc)	$\hat{x}$ $F_{1}$-Score${}_{\mu }$	*σ* $F_{1}$-Score${}_{\mu }$	7% ($F_{1}$)	15% ($F_{1}$)	30% ($F_{1}$)
**1**	**Artificial Hydrocarbon Networks**	**0.9343**	**0.0398**	**0.9696**	**0.9547**	**0.8786**	**0.9697**	**0.0162**	**0.9839**	**0.9781**	**0.9470**
2	*k*-Nearest Neighbors	0.9031	0.0334	0.9059	0.9425	0.8608	0.9478	0.0178	0.9484	0.9692	0.9257
3	SVM with Radial Basis Function Kernel	0.8856	0.0575	0.9276	0.9249	0.8043	0.9426	0.0266	0.9607	0.9621	0.9051
4	Naive Bayes	0.8583	0.0597	0.7769	0.9182	0.8798	0.9313	0.0361	0.8810	0.9638	0.9491
5	Stochastic Gradient Boosting	0.8363	0.1125	0.9722	0.8402	0.6967	0.9284	0.0475	0.9868	0.9278	0.8705
6	SVM with Linear Kernel	0.7981	0.0887	0.8796	0.8400	0.6747	0.8970	0.0400	0.9322	0.9177	0.8410
7	AdaBoost	0.7736	0.1163	0.9151	0.7755	0.6302	0.8970	0.0496	0.9582	0.8960	0.8367
8	Mixture Discriminant Analysis	0.7629	0.1136	0.8816	0.7975	0.6098	0.8693	0.0641	0.9347	0.8909	0.7823
9	Random Forest	0.7612	0.1762	0.9825	0.7498	0.5514	0.9095	0.0625	0.9909	0.8986	0.8390
10	Rule-Based Classifier	0.7465	0.1629	0.9484	0.7414	0.5496	0.8893	0.0680	0.9747	0.8851	0.8082
11	C4.5 Decision Trees	0.7367	0.1695	0.9459	0.7335	0.5308	0.8826	0.0741	0.9739	0.8815	0.7925
12	Linear Discriminant Analysis	0.7089	0.1212	0.8339	0.7478	0.5449	0.8368	0.0700	0.9069	0.8622	0.7412
13	Multivariate Adaptive Regression Splines	0.6756	0.1661	0.8755	0.6824	0.4688	0.8590	0.0710	0.9469	0.8569	0.7731
14	Nearest Shrunken Centroids	0.6422	0.0243	0.6202	0.6761	0.6302	0.7807	0.0231	0.7493	0.8044	0.7883
15	Artificial Neural Networks	0.5864	0.1408	0.7820	0.5214	0.4559	0.7469	0.0864	0.8691	0.6892	0.6824
	**Average**	**0.7740**	**0.1055**	**0.8811**	**0.7897**	**0.6511**	**0.8858**	**0.0502**	**0.9332**	**0.8922**	**0.8321**

**Table 21:** Training and testing times of the supervised classifiers in the complete and the reduced datasets.

No.	Method Name	Training Time (s)		Testing Time (ms)	
		Complete Dataset	Reduced Dataset	Complete Dataset	Reduced Dataset
1	AdaBoost	20.39	6.19	0.55	0.60
**2**	**Artificial Hydrocarbon Networks**	**72.61**	**61.53**	**1.71**	**0.92**
3	C4.5 Decision Trees	2.23	0.91	0.03	0.02
4	*k*-Nearest Neighbors	6.87	3.26	0.60	0.21
5	Linear Discriminant Analysis	10.23	0.13	0.04	0.01
6	Mixture Discriminant Analysis	7.23	5.02	0.21	0.15
7	Multivariate Adaptive Regression Splines	40.26	7.72	0.07	0.03
8	Naive Bayes	29.30	5.76	56.55	11.04
9	Nearest Shrunken Centroids	0.08	0.08	0.01	0.01
10	Artificial Neural Networks	18.29	12.14	0.02	0.01
11	Random Forest	24.73	8.97	0.03	0.04
12	Rule-Based Classifier	3.62	1.19	0.03	0.02
13	Stochastic Gradient Boosting	16.54	5.48	0.07	0.07
14	SVM with Linear Kernel	3.51	3.91	0.10	0.07
15	SVM with Radial Basis Function Kernel	26.20	36.38	1.90	3.02

## Appendix A. Artificial Hydrocarbon Networks: A Numerical Example

This section aims to show training and testing procedures in artificial hydrocarbon networks (AHN) for classification purposes. To this end, a numerical example with a general purpose was elected.

### Appendix A.1. Training Step

Consider that there is a dataset of 20 samples with three features and one label, as shown in Table A1. If an artificial hydrocarbon network model is required, then the training process will be as follows: (i) define the training set; (ii) determine configuration parameters; (iii) run Algorithm 1; and (iv) obtain the AHN-model.

**Table A1:** Dataset used in the numerical example.

No. Sample	$x_{1}$	$x_{2}$	$x_{3}$	*y*		No. Sample	$x_{1}$	$x_{2}$	$x_{3}$	*y*
1	4.3213	3.6221	5.0002	1		11	6.9039	4.9760	3.3688	2
2	4.2141	2.3912	6.8321	1		12	7.3675	2.3451	3.5782	2
3	4.3803	6.7115	6.8618	1		13	7.4727	4.6783	−4.3858	2
4	3.6646	3.5900	6.4806	1		14	7.7866	5.0108	3.4829	2
5	3.2687	6.6652	6.3268	1		15	6.9858	6.8510	0.7049	2
6	3.6156	4.5413	6.6895	1		16	6.8830	15.7787	−3.0057	3
7	3.3436	5.9303	6.4620	1		17	7.5271	17.8117	−3.1938	3
8	4.4932	3.3761	5.4943	1		18	7.7992	16.9416	−2.3485	3
9	4.8778	2.0918	5.5466	1		19	6.9773	16.6224	−2.2307	3
10	4.9102	5.1102	5.4166	1		20	7.0529	17.0954	0.4050	3

#### Appendix A.1.1. Define the Training Set

For this particular example, the training set is defined to be 50% of the original dataset (Table A1), and the remaining 50% will be considered for testing. Random selection is applied. For example, the following samples are part of the training set: {1,5,6,7,13,14,15,16,18,19}.

#### Appendix A.1.2. Determine Configuration Parameters

Three configuration parameters are required for training an AHN-model: the number of molecules *n* in the hydrocarbon compound, the learning rate *η* and the tolerance value ϵ>0. For this case, the selected values are: n=3, η=0.1 and ϵ=0.0001.

#### Appendix A.1.3. Run AHN-Algorithm

Next, Algorithm 1 is computed. Following the algorithm, the first step is to initialize an empty hydrocarbon compound AHN={}. Then, a saturated compound *C* is created using the number of molecules *n* and Equation (2), i.e., $C=CH_{3}-CH_{2}-CH_{3}$. In fact, the latter means that the first molecule has three hydrogen values; the second one has two hydrogen values; and the third one has three hydrogen values. Then, three intermolecular distances $r_{t}$ for t=1,...,n are randomly created. For this example, Table A2 shows the initial intermolecular distances. Notice that each intermolecular distance is a vector in the feature space, such that $r_{t}={\left\{r_{1},r_{2},r_{3}\right\}}_{t}$.

**Table A2:** Summary of the first update of intermolecular distances.

*t*	$r_{t}\left(i=0\right)$	$L_{t}\left(i=0\right)$	$r_{t}\left(i=1\right)$
1	(−0.04, 0.33, −0.01)	(3.27, 3.23, 3.56, 3.54)	(0.11, 0.48, 0.14)
2	(0.36, 0.53, 0.71)	(3.62, 3.98, 4.51, 5.22)	(0.16, 0.33, 0.51)
3	(0.06, 0.14, 0.15)	(−4.39, −4.32, −4.19, −4.04)	(0.21, 0.29, 0.30)

Then, a loop starts until a tolerance criterion is true. Inside this loop, the set of bounds $L_{t}$ is computed using Equation (A1); where $L_{0}$ is the minimum value of each feature in the training set, i.e., $L_{0}\;=\;\left(3.2687,3.6221,-4.3858\right)$. Table A2 shows the first iteration of bounds.

(A1) $$L_{t}=L_{t-1}+r_{t}$$

Then, these bounds define a subset of samples for each molecule. In fact, a subset of samples is used to compute the hydrogen $H_{i}$ and carbon *σ* values in the specific molecule, using Equation (1) and an optimization process. In this work, the least square estimates were used as suggested in [27]. Using these parameters, the compound *C* is built using Equation (3). A prediction with this compound is done in order to calculate the energy of molecules. In this example, the mean squared error is employed. It is remarkable to say that the rounding function was employed at the output of the predicted values. Lastly, the updated values of intermolecular distances are computed using Equations (4) and (5). For this example, assume that the energy values of the molecules are $E_{1}=1.5,E_{2}=-0.5,E_{3}=1.0$ with a steady state $E_{0}=0.0$; then, the intermolecular distance differences are $\Delta r_{1}=0.15$, $\Delta r_{2}=-0.20$, $\Delta r_{3}\;=\;0.15$, and the updated intermolecular distances are those summarized in Table A2.

Once the loop stops, the artificial hydrocarbon network AHN is completed with the following information: the set of CH-molecules, the hydrogen and carbon values of each molecule and the complete set of bounds, such that AHN={C,ψ(x)}. Table A3 summarizes the parameters of the resultant AHN-model.

**Table A3:** Parameters obtained after training the AHN-model.

Parameters	Values
$H_{i}$	(0.0, 0.0, 10.94; 0.0, 0.0, 26.99; 0.0, 0.0, 0.0)
	(0.0, 0.0; 0.0, 16.02)
	(0.0, 0.0, 10.94; 0.0, 0.0, 26.99; 0.0, 0.0, 0.0)
*σ*	(1, 1, 0)
*L*	(3.27, 3.38, 3.86, 3.99)
	(3.62, 3.78, 4.11, 4.62)
	(−4.39, −4.17, −3.89, −3.59)

Notice that the order of molecules is defined by the algorithm; but once the AHN-model is trained, the order has to remain constant when testing.

### Appendix A.2. Testing Step

Once the AHN-model is trained, the testing step considers validating the output predictions of the classifier. In that sense, the testing set is required. Following with this example, the testing set is composed of the samples: {2,3,4,8,9,10,11,12,17,20}. Then, the functional ψ(x) with parameters equal to the ones as shown in Table A3 is used. The inputs of this function are the features of the testing set. For instance, consider the first sample in the testing set x=(4.2141,2.3912,6.8321). This value is tested in ψ(x), which calculated the value ψ(x)=1. As noted, the result is the same as the label. Table A4 shows a comparison between the predicted values using the AHN classifier and the target values. For an extended description of training and testing artificial hydrocarbon networks, see [27,28].

**Table A4:** Comparison between the predicted values $y_{AHN}$ and the target values y.

No Sample	*y*	$y_{AHN}$
2	1	1
3	1	1
4	1	1
8	1	1
9	1	1
10	1	1
11	2	2
12	2	2
17	3	3
20	3	3
